# Utilization of a structured research site mentorship model to facilitate site performance in a clinical research network

**DOI:** 10.1016/j.conctc.2024.101423

**Published:** 2024-12-31

**Authors:** Marcus R. Johnson, Danielle Beck, Melyssa Sueiro, Makaila Decker, Jeff Newcomb, Margaret Tiktin, Amelia Kiliveros, Aliya Asghar

**Affiliations:** a Durham VA Health Care System, USA; b VA San Diego Healthcare System, USA; c VA Miami Healthcare System, USA; d VA Boston Healthcare System, USA; e VA Nebraska-Western Iowa Health Care System, USA; f VA Northeast Ohio Healthcare System, USA; g VA Bronx Healthcare System, USA; h VA Long Beach Healthcare System, USA

**Keywords:** Department of veterans affairs, CSP, NODES, Clinical research, Research site mentorship, Site performance

## Abstract

**Background:**

Research site mentorship has a positive impact on study enrollment. The VA Cooperative Studies Program's (CSP) Network of Dedicated Enrollment Sites (NODES) utilized an existing site mentorship model to onboard 13 new expansion sites. We describe the successes, challenges, and lessons learned during the development and implementation of this model in this paper.

**Methods:**

NODES established a “Site Mentorship/Expansion Workgroup (SWG)” in October 2020 to plan and guide the consortium on providing mentorship and services to other clinical research networks, non-Node CSP study sites, and NODES expansion sites. In 2021, the SWG developed a 12-month implementation plan to onboard 13 new sites by pairing original Node (mentor) sites with expansion Node (mentee) sites. Mentors offered prompt guidance and solutions to mentees on site-level challenges by working with them closely. Implementation of the plan occurred from February 2022 through September 2023.

**Results:**

Data from the implementation of this mentorship plan demonstrated a 32.7 % increase (from 54.8 % in 2022 to 87.5 % in 2023) in the expansion sites’ achievement of their Objectives & Key Results (OKRs). From October 2020–September 2021, prior to mentorship assignments, the original sites (mentors) achieved an average of 88 % of their OKRs and attained an average of 86.7 % and 80.9 % of those OKRs in October–September of 2022 and 2023 respectively during the mentorship implementation phase.

**Conclusions:**

The results demonstrate that developing and implementing a research site mentorship model to facilitate onboarding and performance of research sites into an established network was feasible and contributed to the success of those sites.

## Abbreviations

(VA): Department of Veterans Affairs
(VHA): Veterans Health Administration
(VAMC): Veterans Affairs Medical Center
(ORD): Office of Research & Development
(CSP): Cooperative Studies Program
(NODES): Network of Dedicated Enrollment Sites
(ADOs): NODES Associate Director – Operations
(FWA): Federal Wide Assurance for the Protection of Human Subjects
(HRMACs): Human Resources Consulting and Management Service
(FY): Fiscal Year

## 1 Introduction

Clinical research site mentorship has been shown to be associated with a positive impact on study participant enrollment [1]. Improvement of research sites’ overall performance as a result of their participation in structured mentorship programs is currently limited in published literature, and the available publications in this area are generally related to mentorship in the context of educational and/or professional development for research staff as opposed to site performance [2]. Furthermore, none of the available literatures describe the use of mentorship to facilitate site performance outside of a single study or initiating new research sites into an established clinical research network [[2], [3], [4], [5], [6], [7]]. This situation contributes to an important knowledge gap that warrants further exploration due to the limited use of mentorship to improve clinical research site performance [[1], [8], [9]].

**Fig. 1:**
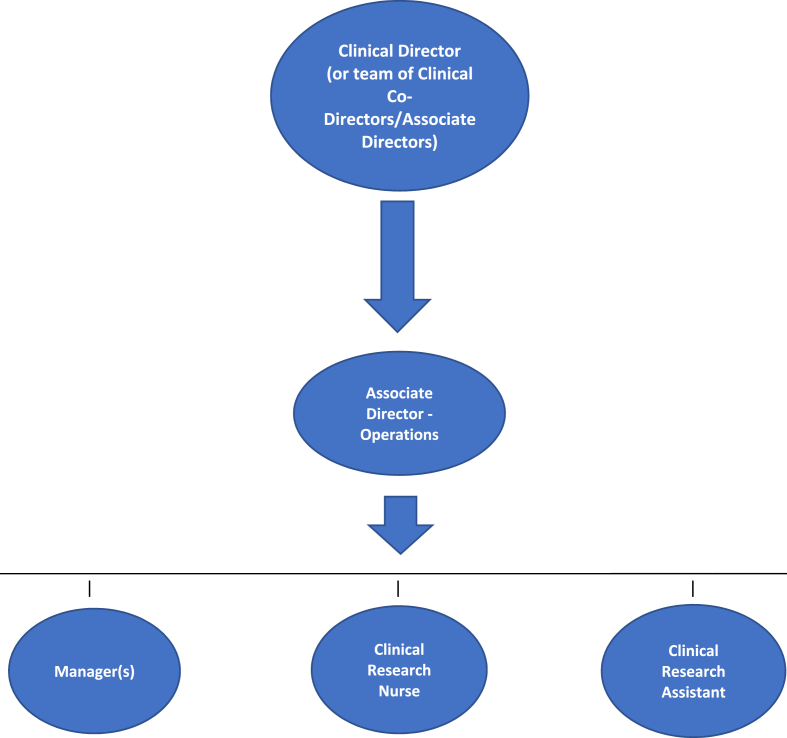
Node site organizational structure.

At the healthcare system level, the work discussed in this manuscript was conducted in the Veterans Health Administration (VHA). VHA is the largest integrated health care system in the United States with 1,321 health care facilities, including 172 VA Medical Centers (VAMCs) and 1138 VA outpatient clinics catering to over 9 million Veterans enrolled in the VA health care program [[10], [11], [12]]. Within VHA, the Department of Veterans Affairs (VA) Cooperative Studies Program (CSP) is a clinical research infrastructure of the VA Office of Research and Development (ORD), and was established to provide coordination for, and enable cooperation on, VA's multi-site clinical trials and epidemiological studies [13,14]. CSP's infrastructure is comprised of several Coordinating Centers responsible for planning and conducting large multi-site clinical trials. Additionally, CSP established a consortium of 23 VAMCs called the Network of Dedicated Enrollment Sites (NODES) that have teams (Nodes) to provide site-level expertise and innovative approaches in addressing challenges to clinical trial execution [[15], [16], [17], [18]]. Each Node site is led by a Clinical Director (or team of Co-Directors), an Associate Director – Operations (ADO), and other clinical research support staff (see Fig. 1). Brief descriptions of these roles can be found in Appendix A [19]. The network started with 10 sites in 2012, and in 2022, it expanded by adding 13 new sites (VAMCs) to the consortium.

Given the NODES program's vast expertise and experience in executing multi-site clinical research within the VA, it is well poised to develop strategies to improve research site performance. Building from the previously established NODES site mentorship model, a mentorship approach was employed with the objective of determining its impact on improving the onboarding process and performance of these new expansion sites into this clinical research network (as measured by their achievement of established NODES performance metrics) [1]. This manuscript describes the successes, challenges, and lessons learned during the development and implementation of a research site mentorship model. We hope this information will serve as a useful reference to other similar networks during expansion efforts.

## 2 Methods

### 2.1 Mentorship plan development

NODES established a “Site Mentorship/Expansion Workgroup (SWG)” in October 2020 aiming to increase the capacity of Node sites to provide mentorship and additional services to other clinical research networks, non-Node CSP study sites, and NODES expansion sites. The SWG was comprised of NODES Directors and ADOs from the 10 original Node sites and NODES/CSP Leadership (CSP Associate Director – NODES). In calendar year 2021, the SWG developed a 12-month mentorship framework with the following components: 1) mentorship plan, 2) performance metrics, 3) mentorship implementation plan, and 4) performance metric evaluation plan. To develop performance metrics, the SWG used the “objectives and key results” (OKRs) tool which is a collaborative goal-setting methodology employed by teams and individuals for setting challenging, ambitious goals with measurable results [20]. These components are outlined in detail in this section and in Appendix C.

The NODES mentorship plan paired original Node (mentor) sites with expansion Node (mentee) sites. Mentors offered prompt guidance and solutions to mentees on site-level challenges related to onboarding into the network, establishing local infrastructure, integrating NODES culture into the local clinical research community and facility, and executing clinical research activities (CSP research) at the site. Thirteen expansion Node sites were selected to participate in the NODES consortium as part of the network's expansion efforts in FY222, while there were only 10 original sites. To ensure that all mentee sites would have access to dedicated mentors, seven of the original sites were paired with expansion sites in a 1:1 ratio and three original sites were paired with two expansion sites each (1:2 ratio) (Appendix D). Several factors including CSP study portfolio overlap, previously established connections between sites, and geographic proximity influenced the site pairing. The mentorship plan included regular virtual meetings over Microsoft Teams® between NODES Directors and ADOs from the mentor and mentee sites. Additionally, the mentorship model incorporated site visits for NODES Directors and ADOs from both mentor and mentee sites. These site visits provided mentor sites an opportunity to directly observe existing challenges, available resources, and potential areas for improvement at the mentee sites, and the mentee sites with a chance to visit their mentor sites and observe their established infrastructure and operations.

### 2.2 Performance metrics (OKR) development (expansion sites)

The SWG created OKRs to assess the performance of expansion sites (Table 1). Two specific OKRs (Objectives 7 & 8) were included to assess performance for the original sites (in the context of site mentorship). An example of these OKRs is referenced below: Objective (2): Raise awareness of research to include progress on current, upcoming, and potential initiatives to facility leadership; Key Result: Expansion sites will meet every other month with their Associate Chief of Staff – Research (ACOS-R) to discuss progress for the first year (post site selection) and then bi-annually; expansion site will meet 3 times per year with their Medical Center Leadership to discuss progress for the first year and then bi-annually;

**Table 1:** NODES expansion site objectives & key results (OKRs) FY22-23.

Expansion Sites (Mentee Sites)	
Objective	Key Results
**1a. Facilitate the dissemination of clinical research best practices across CSP study teams, promote collaboration and foster a culture of “CSP community” at the facility level, and provide study teams with information on the progress of current studies and information on upcoming studies.**	ADOs (and Directors if possible) will meet at least once a month with their existing “all” CSP groups (SCs/RAs should attend these meetings monthly; LSIs/Co-Is should attend these meetings “at least” on a quarterly basis but attendance is encouraged for all meetings)
**1b. Facilitate the dissemination of clinical research best practices across CSP study teams, promote collaboration and foster a culture of “CSP community” at the facility level, and provide study teams with information on the progress of current studies and information on upcoming studies.**	NODES team (Director + ADO) will have at least once a month “one on one” meeting with each CSP team (LSI + SC/RA should be present)
**2a. Raise awareness of research to include progress on current, upcoming, and potential initiatives to the facility leadership.**	NODES Team (Director + ADO) will meet every other month with their ACOS/R to discuss their progress for the first 12 months and bi-annually afterwards
**2b. Raise awareness of research to include progress on current, upcoming, and potential initiatives to the facility leadership.**	NODES Team (Director + ADO) will meet three times a year with their Medical Center Leadership (Director & Chief of Staff and/or their designee) to discuss their progress for the first 12 months and bi-annually afterwards
**3. Increase the capacity of Node sites to be able to provide additional services to other clinical research networks, non-Node CSP study sites, and meet the demands of serving as the leaders of clinical research in VHA.**	NODES Directors and ADOs will visit their mentoring sites as specified in the proposal (these visits will be either in-person or virtual as conditions permit) (Appendix B. Item 3ii)∗∗
**4. Review quality data against defined criteria to evaluate the performance at a Node site for each CSP study, at a Node site for all CSP studies, and for the entire NODES program**	NODES Team (Director + ADO) will attend CSPCC Quality Framework training/presentation within their first 3 months of onboarding (or first 3 months after ADO hire)
**5. Discuss study progress, problem solve issues related to challenges with study execution, and discuss strategy related to the operation of the local Node site.**	NODES Team (Director + ADO) will meet twice per month (preferably weekly)
**6a. Participate in the dissemination of best practices, knowledge sharing, strategic planning, and networking with other colleagues in the NODES program.**	NODES Director will attend 9/12 (75 %) of NODES Program Calls (at a minimum)
**6b. Participate in the dissemination of best practices, knowledge sharing, strategic planning, and networking with other colleagues in the NODES program.**	NODES ADOs will attend 10/12 (83 %) of NODES ADO's calls (at a minimum) (should also aim to attend as many NODES Program Calls as possible)
**Original Sites (Mentor Sites)**	
**7. Increase the capacity of Node sites to be able to provide additional services to other clinical research networks, non-Node CSP study sites, and meet the demands of serving as the leaders of clinical research in VHA.**	≥80 % of the meeting frequency goal as specified in the mentorship plan (item 2) will be met by the mentoring NODES Directors and NODES ADOs
**8. Increase the capacity of Node sites to be able to provide additional services to other clinical research networks, non-Node CSP study sites, and meet the demands of serving as the leaders of clinical research in VHA.**	NODES Directors + ADOs will visit their mentoring sites as specified in the NODES Expansion Site Mentorship Plan (Appendix B. Item 3i)

Performance metrics for the established OKRs were evaluated on a quarterly basis via reports submitted from expansion sites to the CSP NODES Executive Committee (EC). These reports were reviewed by the NODES EC and CSP NODES Leadership who provided suggestions for remediation (if required) and direct acknowledgement of successes to expansion sites. Best practices identified in these reports were disseminated to all sites within the consortium for diffusion. Based on the evaluation data, sites either met, partially met, or did not meet the established OKRs. Additional guidance was provided to expansion sites to enhance their performance if they failed to meet performance metrics in one or more categories.

All sites (expansion and original) were presented with three questions regarding the mentor/mentee experience which included what worked, what did not, and what else could have been helpful. Mentor and mentee sites worked together to present their individual findings at an in-person annual meeting (August 2023) and that data was collated for the analysis. The qualitative data from those presentations were reviewed and analyzed to identify major themes that were identified across multiple mentor-mentee site pairs, for the purpose of this manuscript and is discussed in the Results section. The expansion site mentorship period concluded in September 2023.

### 2.3 Performance metrics (OKR) (original sites)

In FY21-22, prior to the start of site mentorship activities and the onboarding of the expansion sites, the original Node sites (n = 10) had 11 OKRs in place to evaluate their performance across several areas including establishing scheduled meetings with key facility stakeholders, attendance at monthly NODES program calls, execution of “pre-audits” for local CSP studies to prepare for regulatory monitoring and auditing visits, and holding regularly scheduled “combined” meetings with CSP Study Coordinators (SCs), Research Assistants (RAs), and Local site Investigators (LSIs) (Table 2). These OKRs were very “process-oriented” as they were prescriptive regarding items such as the frequency and specific attendees of certain required meetings. In FY23, the NODES EC voted to transition the OKRs to focus on the achievement of actual outcomes as opposed to completing various processes (i.e., revising them to an “outcome-oriented” format). This conversion provided sites with the flexibility to identify and employ strategies of their choice to achieve OKRs, rather than mandating their use of “pre-identified” strategies that were outlined in the previous OKR version. The revised OKRs facilitated and documented the specific and tangible impact (outcomes) of the program's performance metrics. As a result, the number of OKRs used to evaluate the original Node sites' performance decreased from 11 (FY21-22) to 9 (FY23) (Table 3).

**Table 2:** NODES original site objectives & key results (OKRs) FY21-22.

Original Sites (Mentor Sites)	
Objective	Key Results
**1a. Raise awareness of research to include progress on current, upcoming, and potential initiatives to facility leadership, and secure support for critical aspects of research execution to include protected time for Local Site Investigators (LSIs) and space allocation.**	NODES Directors will hold regularly scheduled meetings with the facility Medical Center Director/designee and/or Chief of Staff on a yearly basis (at a minimum), preferably bi-annually (per VA Fiscal Year)
**1b. Raise awareness of research to include progress on current, upcoming, and potential initiatives to facility leadership, and secure support for critical aspects of research execution to include protected time for Local Site Investigators (LSIs) and space allocation.**	NODES Directors will hold regularly scheduled meetings with the facility Associate Chief of Staff – Research (ACOS/R) on a quarterly basis (per VA Fiscal Year)
**2. Raise awareness of current, upcoming, and potential initiatives to the facility Research & Development (R&D) committees. These updates also enhance communication with an important stakeholder to CSP research at the facility-level.**	NODES Directors will hold regularly scheduled meetings with the facility Research & Development (R&D) committees on a bi-annual basis (per VA Fiscal Year)
**3. Allow Directors to participate in the dissemination of best practices, knowledge sharing, strategic planning, and networking with other colleagues in the NODES program.**	NODES Directors will have a 75 % attendance rate at monthly NODES Directors meeting, at a minimum (9/12 months per VA Fiscal Year)
**4. These meetings facilitate the dissemination of clinical research best practices across CSP study teams, promote collaboration and foster a culture of “CSP community” at the facility level, and provide study teams with information on the progress of current studies and information on upcoming studies.**	NODES ADOs will hold regularly scheduled “combined” meetings with Study Coordinators/Research Assistants from ALL CSP study teams collectively on a bi-annual basis (per VA Fiscal Year)
**5. Promote the value of high-quality trial data and decrease the likelihood of deficiencies in trial quality such as protocol deviations, accurate data collection form completion, etc.**	NODES ADOs will provide Oversight and execution of pre-audits with CSP study teams prior to site monitoring and auditing visits by the CSP Site Monitoring, Auditing, and Resource Team (SMART)
**6. Discuss study progress, problem solve issues related to challenges with study execution, and discuss strategy related to the operation of the local Node site; have dedicated time for communication between the ADO and Director is critical to ensure the success of the Node site, including CSP study operations at the site-level.**	NODES ADOs will have regularly scheduled meetings with their NODES Director(s) on a bi-monthly basis (at a minimum), preferably weekly (per VA Fiscal Year)
**7. Participation on national program efforts promotes staff engagement and promotes collaboration not only amongst NODES personnel, but also between NODES and other entities across CSP e.g., CSPCCs, CSPECs, CSP subdomains, etc. Increasing the experience of staff on program-level initiatives should increase their awareness of NODES, CSP, and VA research priorities/initiatives and having that context and knowledge will be beneficial for those involved with research operations at the site-level.**	NODES ADOs will participate on a national program effort, e.g., Strategic Plan Workgroup, Program Committees, participation in manuscript development, etc., on a bi-annual basis (at a minimum) (per VA Fiscal Year)
**8a. Decreasing study start-time expedites improved healthcare for Veterans, accelerates the translation of research findings into clinical practice, and facilitates the likelihood of a study staying within its allocated budget which allows limited funds to be used in other areas of the CSP and VA research enterprise.**	**Timepoint #1** - Start: Local site receives CIRB approval; End: Local site sends R&D approval and supporting documents to CSPCC **Duration/Ranking:** This activity should be completed within 60 days (start to end). Node sites should be in the “upper half” (i.e., shorter time) as compared with non-NODES sites (on average)
**8b. Decreasing study start-time expedites improved healthcare for Veterans, accelerates the translation of research findings into clinical practice, and facilitates the likelihood of a study staying within its allocated budget which allows limited funds to be used in other areas of the CSP and VA research enterprise.**	**Timepoint #2** - Start: Local site receives approval memo from CSPCC to recruit study participants; End: 1st participant enrolled into study **Duration/Ranking:** This activity should be completed within 60 days (start to end). Node sites should have a shorter “first patient in” recruitment time than non-Nodes sites
**9. Improve the feasibility of CSP study protocols that are implemented in the VHA healthcare system, and subsequently improve the likelihood of their success.**	Node sites (Directors and/or ADOs) will participate in at least one CSP study protocol feasibility review annually (at a minimum) (per VA Fiscal Year)

**Table 3:** NODES original site objectives & key results (OKRs) FY23.

Original Sites (Mentor Sites)	
Objective	Key Results
**1. Raise awareness of research to include progress on current, upcoming, and potential initiatives to the facility leadership.**	Each Node site will be able to identify 3–4 examples of facility support (HR/staffing support, space allocation, other type of resource allocation, etc.) that was provided to them in FY23 (Per VA Fiscal Year)
**2. Participate in the dissemination of best practices, knowledge sharing, strategic planning, and networking with other colleagues in the NODES program.**	NODES Directors will attend regularly scheduled monthly NODES Directors calls (At Minimum, 9/12 months (75 %), preferably attend each month's call (100 %)
**3. Facilitate the dissemination of clinical research best practices across CSP study teams, promote collaboration and foster a culture of “CSP community” at the facility level, and provide study teams with information on the progress of current studies and information on upcoming studies.**	At every Node site, each CSP study team will be able to identify 1–2 best practices that they learned/implemented from another CSP study team and/or the respective NODES team (Per VA Fiscal Year)
**4. Promote the value of high-quality trial data and decrease the likelihood of deficiencies in trial quality such as protocol deviations, accurate data collection form completion, etc.**	Provide oversight and execution of SMART pre-audits with CSP study teams
**5. Discuss study progress, problem solve issues related to challenges with study execution, and discuss strategy related to the operation of the local Node site.**	Hold regularly scheduled formal meetings with NODES Director (At Minimum bi-monthly, preferably weekly/per VA Fiscal Year)
**6. Promotion of staff engagement and collaboration not only amongst NODES personnel, but also between NODES and other entities across CSP e.g., CSPCCs, CSPECs, CSP subdomains, etc.**	Participate on national program efforts e.g., Strategic Plan Workgroup, Program Committees, participation in manuscript development, etc. (At Minimum Bi-Annually)
**7a. Accelerate the translation of CSP research findings into clinical practice and facilitate study operational efficiency.**	**Timepoint #1** - The time between when local sites receive CIRB approval and when local sites send R&D approval and supporting documents to CSPCC will be “no greater" then 60 days start to end
**7b. Accelerate the translation of CSP research findings into clinical practice and facilitate study operational efficiency.**	**Timepoint #2** - The time between when local sites receive approval memo from CSPCC to recruit study participants and when 1st participant is enrolled (date consented) into study will be no greater than 60 days start to end
**8. To improve the feasibility of CSP study protocols that are implemented in the VHA healthcare system, and subsequently improve the likelihood of their success.**	Each Node site will review one protocol (per VA Fiscal Year)

## 3 Results

The impact of the site mentorship model on sites’ performance was primarily evaluated during its pre (original sites) and post (original and expansion) implementation, as measured by their achievement of the OKRs. Qualitative data was collected from sites on what they perceived to be the strengths and weaknesses of the model, as well as any significant lessons learned.

### 3.1 Performance metrics (OKR) summary (expansion sites)

Expansion site OKRs were evaluated across FY22 (October 2021–September 2022), and FY23 (October 2022–September 2023). In FY22 and FY23, the expansion Node sites achieved an average of 54.8 % and 87.5 % of their OKRs, respectively. These data represent a 32.7 % increase in the expansion Node sites’ achievement of their OKRs between FY22-23 (Table 4).

**Table 4:** NODES OKR performance data (expansion sites) FY22-23.

Expansion Node Sites (n = 13)	FY22^a^	FY23	% Change
Site K	6/9 (67 %)	9/11 (82 %)	15 %
Site L	3/9 (33 %)	7/11 (64 %)	31 %
Site M	6/9 (67 %)	10/11 (91 %)	24 %
Site N	6/9 (67 %)	11/11 (100 %)	33 %
Site O	5/9 (56 %)	11/11 (100 %)	44 %
Site P	5/9 (56 %)	9/11 (82 %)	26 %
Site Q	7/9 (78 %)	9/11 (82 %)	4 %
Site R	1/9 (11 %)	11/11 (100 %)	89 %
Site S	5/9 (56 %)	9/11 (82 %)	26 %
Site T	7/9 (78 %)	11/11 (100 %)	22 %
Site U	7/9 (78 %)	11/11 (100 %)	22 %
Site V	3/9 (33 %)	9/11 (82 %)	49 %
Site W	3/9 (33 %)	8/11 (73 %)	40 %
	**54.8 %**	**87.5 %**	**32.7 %**

a Does not include the following 2 OKRs:NODES Team (Director + ADO) will attend CSPCC Quality Framework training/presentation within their first 3 months of onboarding (or first 3 months after ADO hire)This training was deferred and provided at an in-person NODES Leadership Meeting (10/26/23–10/27/23) and meeting attendees received credit for the training.NODES Directors + ADOs will visit their mentoring sites as specified in the proposal (item 3i)These site visits were dependent on travel funding, approval from CSP Central Office, and the availability of participants which in some cases led to these sites visits being outside of the pre-determined timeframe (within 3–6 months of NODES ADO onboarding).

### 3.2 Performance metrics (OKR) summary (original sites)

Original site OKRs were evaluated across FY21, FY22, and FY23. In FY21, prior to site mentorship implementation, 88 % of OKRs were achieved on average. In FY22 and FY23, the OKR achievement average was 86.7 % and 80.9 %, respectively. These data represent a decrease of 1.3 % in the original Node sites’ attainment of their OKRs between FY21-22, and an overall decrease of 7.1 % between FY21-FY23 (Table 5).

**Table 5:** NODES OKR performance data (original sites) FY21-23.

Original Node Sites (n = 10)	FY21	FY22	FY23	% Change
Site A	9/11 (82 %)	10/11 (91 %)	7/8 (88 %)	6 %
Site B	10/11 (91 %)	10/11 (91 %)	6/8 (75 %)	(-16 %)
Site C^b^	10/11 (91 %)	9/10 (90 %)	7/8 (88 %)	(-3%)
Site D^b^	10/11 (91 %)	9/10 (90 %)	5/8 (63 %)	(-28 %)
Site E^b^^,^^d^	11/11 (100 %)	9/10 (90 %)	7/7 (100 %)	0
Site F^a^^,^^b^	7/10 (70 %)	7/10 (70 %)	7/8 (88 %)	18 %
Site G^b^^,^^d^	8/11 (73 %)	9/10 (90 %)	4/7 (57 %)	(-16 %)
Site H	11/11 (100 %)	7/11 (64 %)	6/8 (75 %)	(-25 %)
Site I	10/11 (91 %)	10/11 (91 %)	8/8 (100 %)	9 %
Site J^c^	10/11 (91 %)	9/9 (100 %)	6/8 (75 %)	(-16 %)
	**88 %**	**86.7 %**	**80.9 %**	**(-7.1 %)**

a FY21 - Timepoint #2 Metric not included as this timepoint crossed over Fiscal Years (FY21-22) and was therefore unable to be evaluated at the end of FY21 (Site F).

b FY22 - Timepoint #2 Metric not included as this timepoint crossed over Fiscal Years (FY22-23) and was unable to be evaluated at the end of FY22 (Site C, Site D, Site E, Site F, Site G).

c FY22 Timepoint #1 & Timepoint #2 metric data was nonexistent because the site did not launch any new studies in FY22 (Site J).

d FY23 Timepoint #2 is not included because these sites did not have any studies in this start-up phase during FY23 (There was only study initiated at these sites in FY23 and its start date was delayed until FY24) (Site E, Site F).

### 3.3 Qualitative site feedback - successes (all sites)

Each mentor and mentee pair (n = 12) presented at an in-person NODES ADO meeting in August 2023 and highlighted key aspects of their mentorship experience. It is important to note that one mentor/mentee site pair was unable to attend the meeting so there were only 12 presentations provided. The presentations yielded the following common themes as contributors to the success of the mentorship program: Meeting Frequency (n = 12), Knowledgeable Mentor Site (n = 9), Prompt Responses to Questions (n = 8), Check-In's with Mentor ADO Outside Scheduled Meetings (n = 7), Open Communication (n = 7), Mentor/Mentee Node Site Visits (n = 6), and Structure of Mentorship Model (n = 4) (Appendix E). Information related to key highlights of these successes is further described below.

The frequency and flexibility of mentor and mentee meetings was found to be beneficial as expansion Node sites developed their respective site programs. One mentee noted that the one-on-one regular meetings “between site ADOs allowed for productive discussions around site specific challenges, successes, and implementation plans.” Mentee sites emphasized that the structure of the scheduled meetings with the mentor site, which included the NODES Directors, provided a good opportunity for “mentee sites to share updates on the growth of their programs, and obtain valuable feedback.” Understanding the mentee site, and its unique situation and infrastructure was found to be important to mentor ADOs. One mentor ADO noted “in understanding our mentee site's unique challenges, goals, and strengths, we were able to provide tailored guidance and support to meet their specific needs, while helping them navigate their program responsibilities more effectively.”

Mentee ADOs noted that the utilization of multiple communication platforms including email, phone, text, and Microsoft Teams™ were useful to stay in touch, obtain expedited answers, and foster open lines of communication. The flexibility of the mentor sites' availability enabled the mentee sites to achieve the meeting frequency OKR. The open communication between mentor and mentee ADOs was critical in addressing difficult topics, such as “how to handle issues with employees, investigators, participant recruitment and enrollment, etc.” Mentee sites noted that prompt mentor site responses to questions facilitated “communication at the site and helped solve site-level problems quickly.” In-person site visits were found to be beneficial for both the mentor and mentee as these visits helped to bolster the relationship between mentor and mentee teams, with one site stating that the “visits fostered relationship building, bonding, and group cohesion.” Furthermore, the timing of these visits in relation to the implementation of the NODES program at the expansion Node sites was emphasized. One site highlighted the importance of the timing of the site visits stating that the mentee “benefited from the in-person site visit to their mentor site early in Year 1. The timing of both visits maximized site implementation efforts.” Another benefit of the site visits was that they provided the opportunity for mentee sites to observe the mentor site's operations, first-hand, which created “personal connections and understanding of the other NODES positions.” The mentorship model and its framework of metrics was beneficial to expansion sites. The model provided a year-long outline of metrics to help facilitate the successful onboarding and establishment of a Node site program. Of note, the one-on-one mentorship pairing was not impacted for the mentor sites that were paired with two expansion Node sites. As one mentor ADO with two expansion Node sites noted, the mentorship of two expansion Node sites allowed the mentor site to learn “from working with both sites. It was a unique experience overall.”

### 3.4 Qualitative site feedback - challenges (all sites)

With numerous successes, there were also several notable challenges related to the mentorship plan identified as common themes: Timing and duration of site visit (n = 6), Human Resources (HR) Delays (n = 4), Mentorship Model Structure (n = 3), CSP Start-Up Delays (n = 2), Prioritization of Site Initiation Tasks (n = 2). Not all mentorship pairings experienced challenges during the mentorship process, but the top two challenges are described below (Appendix F). Several mentorship pairs noted that the timing and duration of the site visits could be adjusted to yield more favorable results (n = 6). As one mentee ADO stated, having the “in-person mentor site visit scheduled shortly after the new ADO is hired could be helpful for a new ADO as they work to build the program at their site.” The on-site visit by the mentor team could help in identifying areas of opportunity for the new site to focus on. Scheduling this site visit earlier in the implementation process would allow the mentor site to initiate “targeted problem-solving assistance,” while clarifying the “expectations of NODES at earlier in-person meetings.”

Human Resources (HR) related delays, including staff on-boarding and hiring, were also reported as challenges experienced by several mentorship pairings (n = 4). In March 2023, the U.S. Department of Veterans Affairs introduced a new HR platform, Human Resources Management and Consulting Service (HRMACS). This transition contributed to delays in position description classification, hiring, and onboarding of research staff. Several ADOs stated that it would have been beneficial to have all Node support positions classified earlier, before the HRMACS transition. Since not all ADOs started at the same time, there was variability in initiating the mentorship effort across the expansion sites.

In summary, the key results from our work include the demonstrated 32.7 % increase in the expansion sites' achievement of their established OKRs between FY22-23 (Table 3), a noted decrease of 7.1 % in the original Node sites’ attainment of their OKRs between FY21-FY23 (Table 5), and several noted strengths (frequency of meetings, knowledge of mentor sites, responsiveness to mentee site questions, etc.) and areas of improvement (timing and duration of site visits, human resources delays, mentorship model structure, etc.) for the mentorship program that were determined through an analyses of collected qualitative data from mentor and mentee site pairs.

## 4 Discussion

Research study sites are an integral part of the broader clinical research ecosystem. It is paramount that a site's participation in clinical trials networks and studies is predicated on the appropriate type of mentorship and training to ensure their success, given the critical nature of their performance as it relates to studies' achievement of established participant enrollment and data quality targets [[21], [22], [23], [24], [25]]. The results of our work demonstrated that the development and implementation of a site mentorship framework to facilitate onboarding and performance of clinical research sites was successful across participating mentee sites. There are several opportunities for improvement in future iterations of this model, as evidenced by the qualitative feedback obtained from our NODES ADOs. However, the noted 32.7 % increase in the expansion sites' achievement of their established OKRs between FY22-23 (Table 3) demonstrated that this framework provides an effective mechanism to facilitate the onboarding of research sites into an established clinical research network, and their subsequent performance.

The model provided a structured approach for expansion sites to obtain tailored, one-on-one feedback from well-established sites within the NODES consortium. The qualitative feedback from mentor and mentee pairs also provided valuable insights into the successes and challenges of the mentorship program. As reported by mentor sites, the most highly rated components of the mentorship plan were Meeting Frequency (100 %), Mentor Site Knowledge (75 %), Responsiveness (67 %), Check-In's with Mentor ADO Outside Scheduled Meetings (58 %), and Open Communication (58 %), in terms of their contributions to the overall mentorship experience and impact on the outcomes under evaluation (OKR achievement).

Potential limitations and impact on our findings may present challenges to its implementation in other settings. The setting in which we executed this strategy is unique. The VA healthcare system is the largest integrated healthcare system in the United States [26,27]. There are no other integrated healthcare systems in the country that are comparable to the VA in terms of available resources and operational capacity (i.e., overall budget, staffing levels, etc.). Several organizational and structural components of the VA healthcare system, and at a more granular level the VA research enterprise, were taken advantage of. During this effort. These include our program's (CSP) use of a centralized Institutional Review Board (VA Central Institutional Review Board) for the multi-site clinical research that it sponsors, as well as a centralized hiring division for research staff which provides a standardized approach for human resource related activities for research staff, e.g., hiring, promotions, benefits, etc. [28,29] While the mentor-mentee site pairs were part of the same integrated healthcare system (VA), it cannot be assumed that all VA facilities have completely heterogenous operations and infrastructure, i.e., operations and structural systems are not necessarily standardized across facilities and the broader healthcare system. To some extent, it could be assumed that this model could be implemented in research networks that are part of other large healthcare systems (federal government, academic, community-based, etc.) due to similar and existing variance in operations and infrastructure across the facilities in their respective networks as well.

Additionally, the mentor NODES ADOs illustrate one of the many unique aspects and benefits of the VA's infrastructure. They served both as mentors for approximately 19 months (February 2022–September 2023) and in parallel continued their primary roles within NODES and CSP. Other institutions and groups may not have the staffing capacity to deploy individuals to serve in mentor roles as a secondary responsibility for that length of time, nor the funding capacity to hire individuals in the mentor role as a primary position. Lastly, the variability across mentor and mentee sites in terms of their infrastructure prior to and during their onboarding into the NODES consortium had an impact on their performance and overall experience with this mentorship program. For example, sites had varying numbers of CSP studies in their research portfolios prior to and during the mentorship period. There were different levels of infrastructure at these sites (e.g., some facilities provided internal funding to support NODES hires in addition to CSP funding), several facilities had dedicated clinical research units, and varying levels of staff turnover (NODES and study staff) during the mentorship period. It is worth noting a slight overall decrease in the mentor sites' achievement of their established OKRs across FY21-23 (7.1 %). This decrease in performance could be related to a variety of reasons including the mentor ADOs having diminished bandwidth due to the provision of support that provided to the mentee sites. All of these influences warrant further exploration to determine potential correlations between site performance and the overall experience of sites in this mentorship model. Therefore, the replication of this position in other settings (healthcare or non-healthcare) may prove to be challenging for other groups that have a desire to replicate this model in their respective organizations.

Considering the limitations, the pilot confirmed several key strengths. The mentorship program successfully integrated participating research sites into a clinical research network, achieved set goals, and improved performance. The use of a structured mentorship program to facilitate the onboarding of research sites into an established clinical research network, and their subsequent performance, has not been previously examined or employed in a system comparable to the VA. Furthermore, it is possible that expansion sites may have been more forthcoming when discussing obstacles to site onboarding and performance with individuals that they considered to be peers (i.e., other participating sites within the clinical trials network), as opposed to program leadership or other entities that attempted to solicit this information.

## Conclusion

In summary, the results demonstrate that the development and implementation of a research site mentorship model to facilitate the onboarding and performance of research sites into an established clinical research network, was feasible and contributed to the success of those sites. Additional work is needed to determine the effectiveness and generalizability of this approach in other types of clinical research networks, e.g., disease-specific (oncology, mental health, etc.), university-based, pharmaceutical, etc., and other settings.

## CRediT authorship contribution statement

**Marcus R. Johnson:** Writing – review & editing, Writing – original draft, Resources, Project administration, Methodology, Conceptualization. **Danielle Beck:** Writing – review & editing, Writing – original draft, Project administration, Methodology, Conceptualization. **Melyssa Sueiro:** Writing – review & editing, Writing – original draft, Project administration. **Makaila Decker:** Writing – review & editing, Writing – original draft, Project administration, Conceptualization. **Jeff Newcomb:** Writing – review & editing, Writing – original draft, Project administration. **Margaret Tiktin:** Writing – review & editing, Writing – original draft, Project administration. **Amelia Kiliveros:** Writing – review & editing, Writing – original draft, Project administration. **Aliya Asghar:** Writing – review & editing, Writing – original draft, Project administration, Methodology, Conceptualization.

## Disclaimer

The views expressed in this article are those of the authors and do not necessarily represent the views of the Department of Veterans Affairs or the government of the United States.

## Funding

The activities reported/outlined here were supported by the Department of Veterans Affairs, VA Office of Research and Development and the Cooperative Studies Program.

## Declaration of competing interest

The authors declare that they have no known competing financial interests or personal relationships that could have appeared to influence the work reported in this paper.
