# Supplementary material for: Utilization of a structured research site mentorship model to facilitate site performance in a clinical research network
Source: Contemp Clin Trials Commun. 2024 Dec 31;44:101423. doi: 10.1016/j.conctc.2024.101423 (PMC11782873; doi:10.1016/j.conctc.2024.101423)
Supplement: Multimedia component 1 [file mmc1.docx]

| **Appendix A. NODES Position Descriptions** | |
| --- | --- |
| **Role** | **Responsibilities** |
| Clinical Director  (or team of Clinical Co-Directors/Associate Directors) | *Provides oversight, leadership and mentorship to local Node and CSP study teams and ensures the successful conduct of CSP studies at the local Node site  *Identifies, mentors and collaborates with prospective and existing Site Investigators  *Works closely with medical center leadership at the site to promote and encourage clinical trial efforts throughout the institution  *Ensures appropriate resources and support for CSP research efforts including space requests, laboratory needs, or study specific needs  *Works with the NODES Associate Director-Operations to develop the Node site budget  *Ensures CSP and NODES research procedures, process improvements, initiatives and projects are successfully executed at the site  *Strengthens connections within the CSP network to provide greater opportunities for interdisciplinary research  *Collaborates with local and national stakeholders to achieve CSP objectives  *Engages with CSP Coordinating Centers in the feasibility, planning, and implementation of CSP trials    *Participate in programmatic strategic planning of CSP and NODES  * Facilitates the submission of CSP study Letters of Intent (LOI) from the site for review and potential funding |
| Associate Director-Operations | *Provides supervision, leadership and mentorship to local Node and CSP study teams  *Identifies, mentors and collaborates with prospective and existing Site Coordinators and other CSP study team members  *Works with the NODES Director to develop the Node site budget  *Works with study team members to develop site study budgets for each site’s respective studies  *Ensures appropriate resources and support for CSP and NODES research efforts  *Ensures research process improvement initiatives and projects are successfully executed  *Strengthens connections within the CSP network to provide greater opportunities for interdisciplinary research  *Collaborates with local and national stakeholders to achieve CSP objectives  *Engages with CSP Coordinating Centers in the feasibility, planning, and implementation of CSP trials  *Participates in programmatic strategic planning of CSP and NODES  *Provides mentorship for new Node Sites/NODES Expansion efforts  *Provides mentorship and support for CSP study team members  *Provides/arranges for back-up coverage for study team members that are on planned and unexpected leave  *Human Resources: Facilitates job posting, interviewing, hiring, and training for study staff (study coordinators, research nurses, study research assistants, etc.)  *Conducts meetings with site study teams to share best practices, deliver education and training, and to discuss successes/challenges as it relates to clinical trial execution  *Completes local and national study auditing, as well as data and adverse event reporting  *Assists with special projects/workgroups locally & nationally |
| Manager(s) | *Provides oversight, direction and guidance to local CSP study teams on all clinical trial related activities  *Collaborates with local and national stakeholders to achieve CSP objectives  *Assists Director and Associate Director-Operations in engagement with CSP Coordinating Centers in the feasibility, planning, and implementation of CSP trials  *Human Resources: Facilitates job posting, interviewing, hiring, and training for study staff (study coordinators, research nurses, study research assistants, etc.)  *Provides back-up coverage for study team members that are on planned and unexpected leave  *Completes local and national study auditing, as well as data and adverse event reporting  *Assists with coordinating and executing meetings with site study teams to share best practices, deliver education and training, and to discuss successes/challenges as it relates to clinical trial execution  *Assists with special projects/workgroups locally & nationally |
| Clinical Research Nurse | *Provides back-up coverage for study team members that are on planned and unexpected leave (for all studies)  *Provides medical informatics expertise as it relates to the Electronic Medical Record  *Forms/Templates/Documents/Poster Specialist  *Assists with special projects/workgroups locally & nationally |
| Clinical Research Administrator | *Provides back-up coverage for study team members that are on planned and unexpected leave (for studies not requiring an RN)  *Coordinates required performance data submissions for program evaluation efforts  *Produces Bi-Annual local NODES Newsletter  *Schedules meetings, prepares agendas, & minutes  *Organizes/Plans/Arranges travel  *Maintains Director’s calendar  *Assists with special projects/workgroups locally & nationally |
| Clinical Research Assistant | * Coordinates monthly meetings with site study teams to share best practices, deliver education and training, and to discuss successes/challenges as it relates to clinical trial execution  * Assists site teams with travel coordination for CSP-related study kick-off/annual meetings  *Assists site teams with CSP related purchase orders  *Assists new hires with completion of VA trainings |
