# Supplementary material for: Utilization of a structured research site mentorship model to facilitate site performance in a clinical research network
Source: Contemp Clin Trials Commun. 2024 Dec 31;44:101423. doi: 10.1016/j.conctc.2024.101423 (PMC11782873; doi:10.1016/j.conctc.2024.101423)
Supplement: Multimedia component 3 [file mmc3.docx]

Mentorship Plan (Year 1): Each site will be assessed on these metrics for 12 calendar months from the date of their R&D approval (dates will vary by site)

WG Members: Alexandra Kantorowicz, Aliya Asghar, Danielle Beck, Eleanor Lederer, Karen Bratcher, Makaila Decker, Marcus Johnson, Sarah Perusich, Tawni Kenworthy-Heinige

**Mentorship Plan:**

1. One current Node site will be assigned to mentor one new Node site*

2. Regular Meetings will be held between the mentoring and the mentee sites. The frequency of

meetings will be as below:

1. Combined introductory meeting will be held between NODES Director(s) and NODES ADOs of both sites within one month of the new Node site selection (*for sites that had identified an ADO within the one month of selection)*
2. NODES Directors from both sites will meet remotely once a month during the first 3 months and quarterly for the next 6 months (additional meetings can be arranged), and then as needed
3. NODES ADOs from both sites will meet remotely twice a month during the first

months and then monthly for the next 6 months (additional meetings can be arranged), and then as needed

3. Site Visits:

1. Mentee NODES Director(s) and ADO will visit the mentoring Node site within the

first 3 months but no later than 6 months of the new site selection and/or within 3 - 6 months of onboarding of the NODES ADO (site visit can be for up to 3 days)**

1. Mentoring NODES Director(s) and ADO will visit the new Node site 1 month to 3 months after the visit of the mentee NODES Director(s) and ADO to their site (site visit can be for up to 3 days)**

4. Plans for NODES Expansion Sites unable to appoint an ADO by the Q3 of FY23 (April 01,

2023)

i. NODES central leadership will meet with the NODES Director(s) of the site and decide the future outcome

5. NODES Expansion sites will be assessed on the NODES enrollment/site performance metrics

from the Q3 of FY23 (April 01, 2023)

6. Plans for new Node sites not meeting study enrollment/site performance metrics

1. NODES can implement its site mentorship plan for a particular study (if the issue is related to a specific CSP study)
2. If the site mentorship plan does not work, the issue will be discussed with the NODES Executive Committee

7. Plans for inviting ADOs from new Node sites to NODES National WGs/Efforts

i. ADOs will be invited to national program WGs starting Q3 of FY23

**Metrics**

**For new Node sites:**

1. ADOs (and Directors if possible) will meet at least once a month with their existing “all”

CSP groups (SCs/RAs should attend these meetings monthly; LSIs/Co-Is should attend these meetings “at least” on a quarterly basis, but attendance is encouraged for all meetings)

2. NODES team (Director + ADO) will have at least once a month “one on one” meeting with each CSP team (LSI + SC/RA should be present) within 3 months after ADO identification/hire

3. NODES Team (Director + ADO) will meet every other month with their ACOS/R to discuss their progress for the first 12 months and bi-annually afterwards

4. NODES Team (Director + ADO) will meet three times a year with their Medical Center Leadership (Director & Chief of Staff and/or their designee) to discuss their progress for the first 12 months and bi-annually afterwards

5. ADOs will visit their mentoring sites as specified in the proposal (these visits will be either in-person or virtual as conditions permit) (item 3ii)**

6. NODES Team (Director + ADO) will attend CSPCC Quality Framework training/presentation within their first 3 months of onboarding (or first 3 months after ADO identification/hire)

7. NODES Team (Director + ADO) will meet twice per month (preferably weekly)

8. NODES Director will attend 9/12 (75%) of NODES Program Calls (at a minimum)

9. NODES ADOs will attend 10/12 (83%) of NODES ADO’s calls (at a minimum) (should also aim to attend as many NODES Program Calls as possible)

**For existing Node sites:**

1. ≥ 80% of the meeting frequency goal as specified in the mentorship plan (item 2) will be met by the mentoring NODES Directors and NODES ADOs

2. NODES ADOs will visit their mentoring sites as specified in the proposal (item 3i)

* *Due to the fact that there were 10 original Node sites, and 13 onboarded expansion sites, 3 of the original Node mentor sites were assigned 2 mentee sites*

*** Pending current VA travel guidance*
