# Supplementary material for: Utilization of a structured research site mentorship model to facilitate site performance in a clinical research network
Source: Contemp Clin Trials Commun. 2024 Dec 31;44:101423. doi: 10.1016/j.conctc.2024.101423 (PMC11782873; doi:10.1016/j.conctc.2024.101423)
Supplement: Multimedia component 4 [file mmc4.docx]

| **Original Node Sites (Mentor Sites)** | **Expansion Node Sites (Mentee Sites)** |
| --- | --- |
| **VA Boston Healthcare System**  *Boston, MA* | **Philadelphia VA Healthcare System**  *Philadelphia, PA* |
| **VA North Texas Health Care System**  *Dallas, TX* | **Miami VA Healthcare System**  *Miami, FL*    **VA Atlanta Healthcare System**  *Atlanta, GA* |
| **Edward Hines, Jr. VA Hospital**   *Hines, IL* | **William S. Middleton Memorial Veterans Hospital**  *Madison, WI* |
| **Michael E. DeBakey VA Medical Center**  *Houston, TX* | **VA Ann Arbor Healthcare System**  *Ann Arbor, MI* |
| **VA Long Beach Healthcare System**  *Long Beach, CA* | **VA Greater Los Angeles Healthcare System**  *Los Angeles, CA*  **VA Northeast Ohio Healthcare System**  *Cleveland, OH* |
| **Minneapolis VA Health Care System**  *Minneapolis, MN* | **VA Nebraska-Western Iowa Health Care System** *Omaha, NE* |
| **VA Palo Alto Health Care System**  *Palo Alto, CA* | **Rocky Mountain Regional VA Medical Center**  *Aurora, CO* |
| **VA Portland Health Care System**  *Portland, OR* | **VA Puget Sound Health Care System**  *Seattle, WA* |
| **VA Salt Lake City Healthcare System**  *Salt Lake City, UT* | **VA Bronx Healthcare System**  *Bronx, NY* |
| **VA San Diego Health Care System**  *San Diego, CA* | **VA Milwaukee Health Care System**  *Milwaukee, WI*  **VA Connecticut Healthcare System**  *West Haven, CT* |
