# Supplementary material for: Utilization of a structured research site mentorship model to facilitate site performance in a clinical research network
Source: Contemp Clin Trials Commun. 2024 Dec 31;44:101423. doi: 10.1016/j.conctc.2024.101423 (PMC11782873; doi:10.1016/j.conctc.2024.101423)

Appendix E. Components of Success for Mentorship Pairings

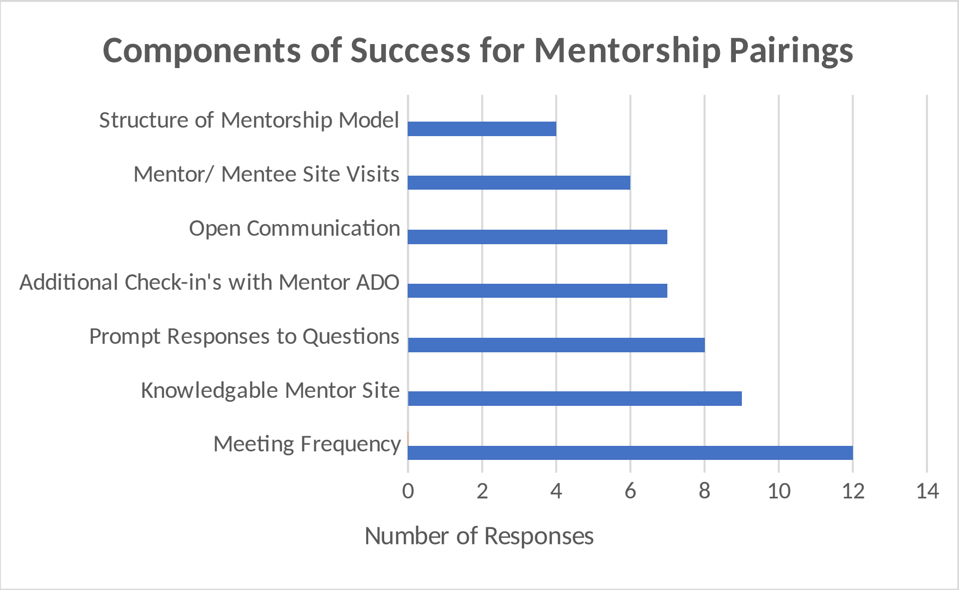

Supplement: Supplementary file 5 [file mmc5.docx]
